# An app-based ecological momentary assessment of undergraduate student mental Health during the COVID-19 pandemic in Canada (Smart Healthy Campus Version 2.0): Longitudinal study

**DOI:** 10.1371/journal.pdig.0000239

**Published:** 2024-05-20

**Authors:** Chris Brogly, Daniel J. Lizotte, Marc Mitchell, Mark Speechley, Arlene MacDougall, Erin Huner, Kelly K. Anderson, Michael A. Bauer

**Affiliations:** 1 Faculty of Information and Media Studies, Western University, London, Canada; 2 Faculty of Health Sciences, Western University, London, Canada; 3 Department of Computer Science, Western University, London, Canada; 4 Department of Epidemiology and Biostatistics, Schulich School of Medicine and Dentistry, Western University, London, Canada; 5 School of Kinesiology, Faculty of Health Sciences, Western University, London, Canada; 6 Department of Psychiatry, Schulich School of Medicine and Dentistry, Western University, London, Canada; 7 Ivey Business School, Western University, London, Canada; Iran University of Medical Sciences, IRAN (ISLAMIC REPUBLIC OF)

## Abstract

This paper presents results from the Smart Healthy Campus 2.0 study/smartphone app, developed and used to collect mental health-related lifestyle data from 86 Canadian undergraduates January–August 2021. Objectives of the study were to 1) address the absence of longitudinal mental health overview and lifestyle-related data from Canadian undergraduate students, and 2) to identify associations between these self-reported mental health overviews (questionnaires) and lifestyle-related measures (from smartphone digital measures). This was a longitudinal repeat measures study conducted over 40 weeks. A 9-item mental health questionnaire was accessible once daily in the app. Two variants of this mental health questionnaire existed; the first was a weekly variant, available each Monday or until a participant responded during the week. The second was a daily variant available after the weekly variant. 6518 digital measure samples and 1722 questionnaire responses were collected. Mixed models were fit for responses to the two questionnaire variants and 12 phone digital measures (e.g. GPS, step counts). The daily questionnaire had positive associations with floors walked, installed apps, and campus proximity, while having negative associations with uptime, and daily calendar events. Daily depression had a positive association with uptime. Daily resilience appeared to have a slight positive association with campus proximity. The weekly questionnaire variant had positive associations with device idling and installed apps, and negative associations with floors walked, calendar events, and campus proximity. Physical activity, weekly, had a negative association with uptime, and a positive association with calendar events and device idling. These lifestyle indicators that associated with student mental health during the COVID-19 pandemic suggest directions for new mental health-related interventions (digital or otherwise) and further efforts in mental health surveillance under comparable circumstances.

## Author summary

This paper analyzes the associations between digital measures from smartphones (such as GPS and step counts) and a broad mental health questionnaire (covering items such as depression and anxiety). This data was collected from students at a relatively large, urban university in Canada during the COVID-19 pandemic. We conducted this study because smartphone-based studies observing aspects of mental health in students as they go about their daily lives are uncommon in Canada. Additionally, mental health concerns, such as depression and anxiety, can be common on university campuses, although it isn’t always clear what is associated with those concerns. We examined an overview of student mental health and how it would relate to digital measures coming from smartphones during the pandemic, as this information would be relevant to inform future pandemics. In general, these relationships might potentially inform ways to improve student mental health, or potentially predict aspects of it based on data coming in from smartphones.

## Introduction

Mental health issues on university campuses are common during periods of traditional in-person study [1]. Universities in Canada [2] have recognized that undergraduate students are faced with a number of stresses that challenge mental health. Undergraduate studies are demanding, and students often feel overwhelmed by their obligations [1]. Additionally, the COVID-19 pandemic had an unprecedented effect on post-secondary students all over Canada, who experienced disruptive [3–7] changes to their studies in response to necessary public health measures regarding COVID-19. Canadian undergraduates have been surveyed regarding aspects of mental health using traditional sampling techniques such as questionnaires and interviews on several occasions [8–10]. Another sampling technique particularly useful to this area is the Ecological Momentary Assessment (EMA) which allows for students to be sampled regarding aspects of their mental health as they go about their day; EMA studies are generally facilitated via mobile apps that run on Android and iOS. In addition to digital questionnaire responses, the apps can collect a wealth of data related to mental health from device hardware, such as GPS location, step counts, and indicators of life activity. For instance, the StudentLife study from Dartmouth College was one of the first in this area to assess student mental health using an app [11]. EMA-based studies on undergraduate mental health are, to the best of our knowledge, limited in Canada. While the undergraduate experience in Canada is comparable to that of other countries, EMA-based studies originating from other countries may not necessarily capture the same experience.

**Fig 1:**
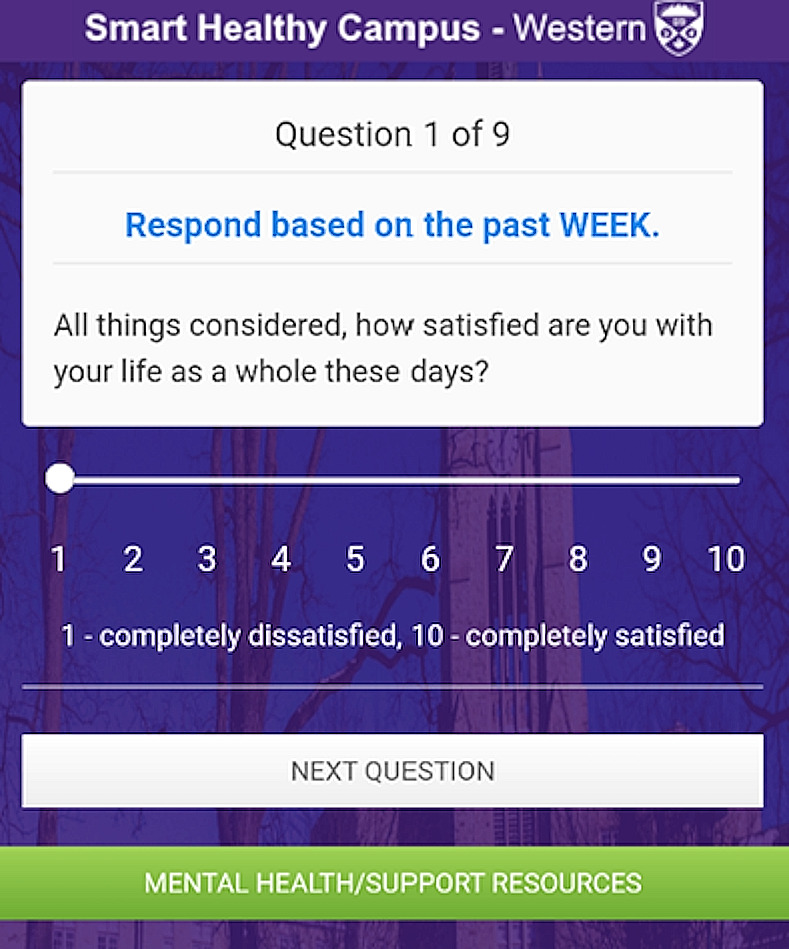
Image of the Smart Healthy Campus 2.0 app during a questionnaire.

We had a particular interest in how mental health measures might be associated with various digital measures coming from smartphone hardware. Although globally there are examples of EMA-type mental health studies in students and others, such as Fried et al. [12], Kleiman et al [13], Schultz et al [14] and Arend et al [15], there does not appear to be a Canadian study directly investigating mental health measures and associations with hardware measures. As a result, we developed and launched the Smart Healthy Campus 2.0 app on Android and iOS. This ran on a research platform we called EMAX, which is fully described in a previous paper [16]. This SHC 2.0 study is the successor to a limited pilot study detailed in a previous paper [17]. The SHC 2.0 app asks a small number of mental health-related questions daily and collects data from phone hardware as questionnaires are submitted, and passively in the background. It was designed to be run during traditional on-campus study for an extended period, such as several months or even semesters. However, this SHC 2.0 study ended up being launched and run during a significant portion of the COVID-19 pandemic. We also launched a separate, strongly-pandemic focused study/app called Student Pandemic Experience (SPE) [18], which collected more data than the SHC 2.0 study, but was not suitable for use past the pandemic due to the specialized questionnaires used. For SHC 2.0, general objectives of the study were to 1) address the lack of longitudinal data in Canada on overviews of student mental health and any potential related measures of lifestyle, and 2) to identify associations between these self-reported overviews of mental health (from questionnaires) and lifestyle-related smartphone digital measures. These objectives were intended to be met by answering the following research question: What associations exist between student psychosocial outcomes (measured by short weekly/daily 9-item mental health questionnaires) and day-to-day behavior (e.g., physical activity, sleep, device usage) during a pandemic? In order to answer this, we focused on providing a more complete analysis of all device digital measures that might relate to student mental health rather than focusing heavily on specific ones. Available smartphone digital measures (such as GPS, step counts) were still categorized into related groups, informed by previous work in mobile sensing [19], including 1) Movement and physical activity, 2) Device usage, and 3) Social and life activity indicators. We analyzed the repeat measures collected via the SHC 2.0 app (shown below in Fig 1) with mixed linear models. The idea behind the mixed model analysis is that by knowing the measurable behaviors/lifestyle associated with positive mental health, it should become easier to design traditional or computer-related interventions to direct participants to those associated behaviors/lifestyle. Another goal is that these models might be integrated into future apps that directly provide interventions or predictions which might be triggered by these models or similar ones.

## Methods

### Informed consent

Consent was obtained from all research participants prior to the commencement of the research.

### Study design

SHC 2.0 was an Ecological Momentary Assessment-type app-based longitudinal cohort study with a repeated measures design. The SHC 2.0 app collected responses to a short questionnaire with a range of data from phone hardware potentially relevant to student mental health; responses and data were sent encrypted to our server when a participant submitted a questionnaire. The SHC 2.0 app also collected this same phone data in the background passively on an hourly basis; participants could set this collection event to 1, 2, or 3 hours. This paper reports on 40 weeks of data collected for SHC 2.0. Participants could download and use the SHC 2.0 app and complete surveys as often or as little as they wanted, as there were no specific requirements on participation. As a result, the total number of participants and samples used in our statistical analysis varies from model to model.

### Ethical approval

Ethics approval was provided by Western University’s Health Sciences Research Ethics Board (HSREB) in October 2020 (Project ID 116670).3

### Recruitment

Mass email recruitment was conducted. All Western University undergraduate students were invited over email to download the SHC 2.0 app in January 2021 and in March 2021 in order to participate in the study. The app was available on Google Play (Android) and the Apple App store. Sign-up information, including demographic information, was collected entirely in the app and stored encrypted. To sign up for the SHC 2.0 app, participants had to view and accept the terms of the letter of information for the study, which listed all data collection procedures. 27000 undergraduate students were emailed. 94 participants signed up for the app, and 86 participants both signed up and did at least one questionnaire in the app. Unfortunately, internet recruitment can have disappointing results [20]. The study participation rate was technically less than 1%. Our institution also regularly recruited for studies across all other disciplines via email.

### Inclusion and exclusion criteria

The only inclusion criterion was that a participant was a full-time undergraduate student at Western University with an iOS/Android phone. Exclusion criteria were students in graduate or professional programs. Participants had to select their undergraduate program from a list in the SHC 2.0 app during the sign-up process.

### Participant compensation

**Fig 2:**
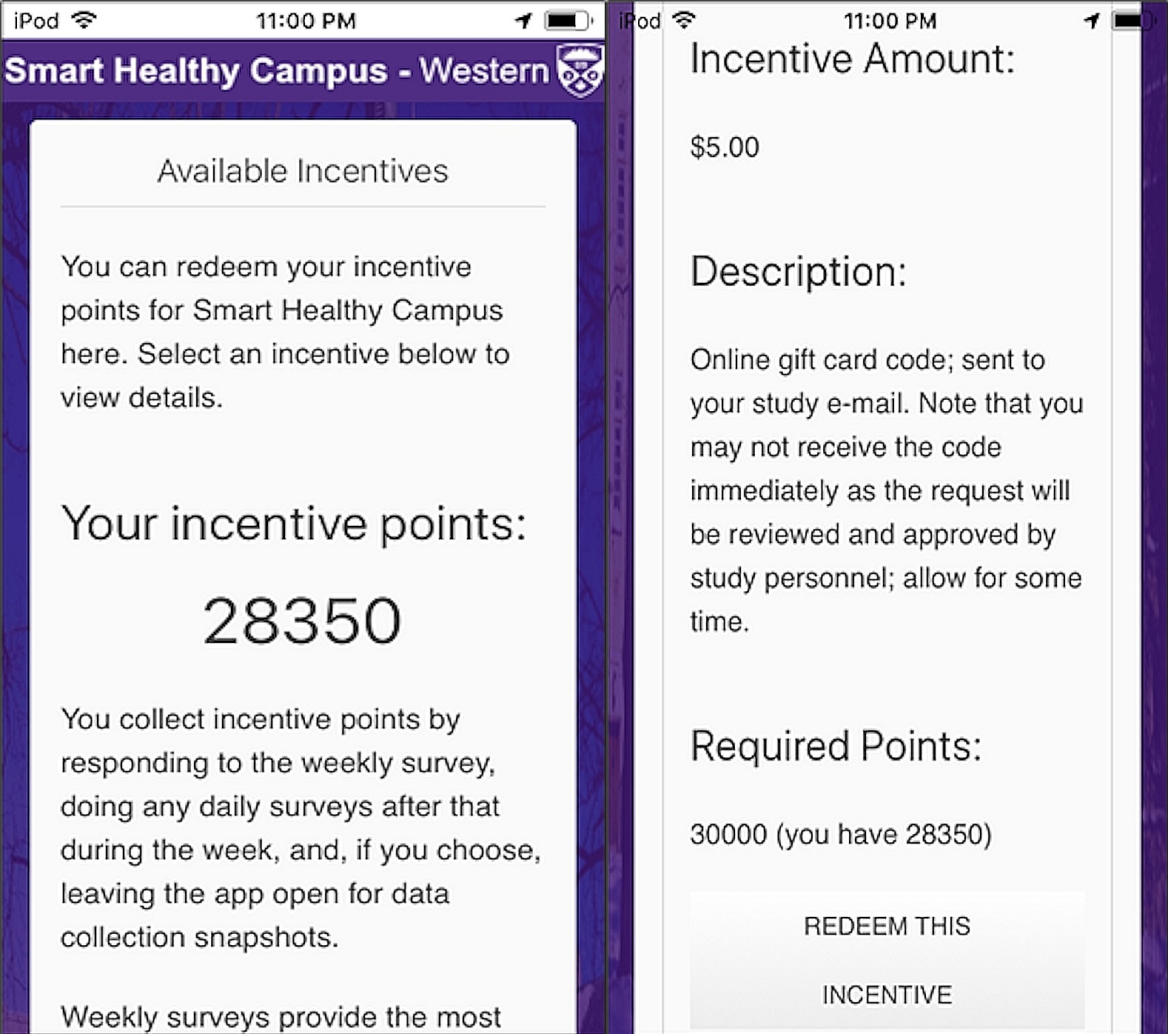
SHC 2.0 point-based incentive system screen (left image). The app has to be scrolled to see the entire screen (right image).

The SHC 2.0 app included a points-based incentive system. Participants could accumulate points through app use and redeem 30000 points for a $5 Amazon gift card. Rewards were offered as the study progressed, and there were no limits on the total amount of gift cards redeemed. The first survey of the week, the weekly survey, would yield 3000 points. Daily surveys would provide 500 points, and passive background data collection events would provide 100. These point values were selected to prevent too much incentivization during a single week. Fig 2 shows an example of using the incentive system. It also allowed for the potential to add new rewards as the study progressed, although only the $5 Amazon gift cards were used during the study.

### Measures–Smart Healthy Campus 2.0 Questionnaire–Details

The SHC 2.0 app always makes a 9-item questionnaire available once per day. However, there are two variants to this questionnaire; the first is the weekly variant, which is available during a new week starting Monday. The weekly variant was designed to capture the experience of the entire past week in case participants were infrequent responders. It would remain open as the first questionnaire to be completed for that week. As an example, if a participant waited until Friday or even Saturday or Sunday to provide a questionnaire response, the weekly variant would still be available then, since that would be the first questionnaire response of that week. If the weekly variant was completed, the daily variant was then available every day until the next Monday, including on Saturday and Sunday. These variants are very similar–only 2 of the 9 questions change and the text of the questions were edited to reflect either the daily or weekly focus. If a participant did not complete at least the weekly variant, then they did not provide any responses at all for that given week, although hourly passive background data may still have been collected by the SHC 2.0 app. Reminders to complete questionnaires were sent to participant devices via standard push notification manually by research personnel, usually at the beginning of the week, and occasionally throughout the week, but a set schedule for reminders was not followed. These notifications were generic reminders for all participants and did not change if a participant had completed a survey for that day. Question topics from both variants cover a range of mental health-related measures relevant to students; these are summarized in Table 1. For instance, two questions on resilience (weekly/daily) were included. The questionnaire topics are described in more detail below. Full questionnaires used in the app can be found in S1 Appendix.

**Table 1:** Mental health domain coverage of the SHC 2.0 weekly and daily questionnaire variants. This table lists the domain of each of the 9 questions and its source. Questionnaires are available in S1 Appendix.

Weekly Variant Mental Health Domain	Source	Daily Variant Mental Health Domain	Source
Life Satisfaction	[21]	Life satisfaction	[21]
Psychological well-being	[22]	Psychological well-being	[22]
Resilience	[23]	Resilience	[23]
Anxiety	[24]	Anxiety	[24]
Depression	[25]	Depression	[25]
Community connectedness	[26]	Community connectedness	[26]
Physical Activity	[27]	Anxiety	[25]
Anxiety	[25]	Psychological Distress	[28,29]
Resilience	[23]	Resilience	[23]

### Measures–Smart Healthy Campus 2.0 questionnaire, weekly and daily variant

The weekly variant was previously shown to significantly correlate with mental health domains based on longer validated full-length questionnaires in a previous pilot study [17]. The daily variant had minor content edits of the first 7 weekly questions to be more reflective of the day.

### Digital measures–overview

From the SHC 2.0 app on the participant devices, 12 digital measures were selected for analysis in this paper (these data came almost exclusively from smartphones but tablets still were occasionally used). There were more than 12 measures collected via the SHC 2.0 app, but these 12 selected for analysis here had the greatest amount of data available across both Android and iOS platforms. A full list of digital measures that the SHC 2.0 app collected, and security measures in-place to protect participants, can be found in our paper on the EMAX platform which powered the SHC 2.0 app [16]. The SHC 2.0 app via EMAX uses heavy RSA-4096 encryption on all data collected from participants. We chose a strong public-key cryptosystem because data decryption keys could be kept offline and secret. This was intended to provide robust protection for sensitive data.

The digital measures were collected during two types of data collection “events”: Active and Passive. Active events occurred whenever a participant responded to a survey. Passive events occurred as a result of ongoing data collection, which happened on a 1, 2, or 3 hour basis (approximately). Participants could use a slider in the app to choose whether passive background data collection should occur approximately every 1, 2, or 3 hours. For both the Active and Passive events, digital measure data were uploaded to our server. The Active event, unlike the Passive event, also contained the response data to a weekly survey. Since Passive events did not have this data, but it was needed for mixed model fits to work, an algorithm was run over all the Passive event data to connect it to a recent Active questionnaire result found before the Passive event occurred. The algorithm would join a questionnaire response to any digital measures data up to the nearest daily questionnaire response, the next weekly questionnaire response, or 3 days, whichever came first.

### Digital measures ‐ movement and physical activity

To determine associations between mental health questionnaire results and movement and physical activity, we examined 4 sources of data. First, we looked at total weekly step count. Step counts are commonly analyzed in mobile sensing studies [30]. Second, we examined GPS locations visited. We define GPS locations visited as a sum of any significant changes in geographic location. Our SHC 2.0 app only detected GPS coordinates to two decimal places, so a “significant change” of at least 0.01 (or about 1.1km) in the latitude/longitude of a coordinate is required to increase this sum. Third, we define floors walked as floors gone up + floors gone down over the past week. Fourth, a location “flag” was used (called “Campus Dist.” in the results section), and it could have three values: (1) on-campus, (2) in London, Canada and (3) outside of London, Canada. Most classes had transitioned to an online format at our institution during the pandemic; some students continued to live on campus while some went home.

### Digital measures ‐ device usage

To determine associations between mental health questionnaire results and device usage, we examined 5 sources of data. First, we collected system uptime in milliseconds. This is defined as the number of milliseconds the device has been powered on for since boot. We also obtained system uptime + deep operating system sleep (when the device is left idle for a period), in milliseconds. Second, we obtained available RAM (available phone Random Access Memory) in bytes. Unlike CPU usage, available RAM (in bytes) is straightforward to collect across iOS/Android platforms. Third, we obtained a percentage of internal free space. Finally, we timed how long users had run the SHC 2.0 app on their device, in milliseconds. Some of these may appear unrelated to mental health, but to the best of our knowledge, we are not aware of any studies definitively proving they should not be considered.

### Digital measures–social/life activity indicators

Contacts and calendars are data sources that may be indicative of social or life activity and they were straightforward to access across platforms. The sum of calendar events were collected. These events may include items like reminders for physical activity, events, and appointments. A sum of all contacts at each sample was also obtained. Additionally, a basic algorithm to check for family-related names (or containing family-related names) in contacts was run to collect a sum of these. Actual names were never transmitted to our server; we only received numeric sums.

### Digital measures ‐ additional item: Number of apps installed

One additional data source was included in this analysis and considered a digital measure. This was a percentage of popular installed apps from a known list of 40. However, we did not split these by category due to the small list of 40.

### Statistical analysis

The focus of the statistical analysis in this paper is on the relationship between phone digital measures and the SHC 2.0 questionnaire variants. The total score of the SHC 2.0 questionnaire variants are examined (some items were recoded to make them suitable for a total sum), along with scores from individual questions, which are representative of various mental health domains [17] (see Methods). SHC 2.0 required participants to answer all questions since the questionnaires were short.

R 4.1.1 “Kick Things” and RStudio were used for all statistics. All mixed linear models shown were constructed using the lmerTest library [31] which provides p-values for effects using the Satterthwaite degrees of freedom method [31]. sjPlot/dwplot were used for coefficient graphs and ggplot2 was used for line graphs. This approach to mixed linear models with EMA data and the same R packages were previously used by Huckins et al. [32] and Mack et al. [33] in their study on student mental health and the impact of COVID-19 at Dartmouth College.

With regards to building the mixed linear models, z-score standardization was used on digital measure items with R’s scale(…) function as values could vary considerably. For each mental health questionnaire (DV), a first model was fit with all 12 previously discussed digital measure items as fixed-effects. The participants were the random-effect. Then, all the digital measure items that lmerTest did not report significance for were removed, and a second, final model was fit with only the significant digital measure items (backward elimination). The reason for fitting and only using a second model with backward elimination was that removing those insignificant terms sometimes greatly increased the amount of complete case digital measure samples available for use in the lmerTest models. The first round of mixed models are found in S2 Appendix. Terms were considered significant if the p-value was less than 0.005. This was value was chosen because it is 0.05 with Bonferroni correction applied for 10 multiple tests. The value 0.005 is used for both daily and weekly questionnaires, even though we only reported on 7 items for the weekly questionnaire compared to 10 for the daily one.

### Data inclusion

Highly correlated mixed model parameters can make each other non-significant even if they are predictive. There were many significant correlations between the parameters used for these models, however, the majority were very weak or weak based on Spearman coefficients (0.0–0.20). Some coefficients did enter moderate territory in the range of 0.4 to 0.6. Additionally, there was a strong correlation between system uptime, and system uptime with deep sleep, although we argue this was acceptable as those likely better highlighted differences between device use and potential idle or sleep time for participants; additionally, more than one model resulted in significance for only one of these parameters, while others produced significance for both, suggesting the correlation was not important. We argue these correlations in our 12 selected digital measure items, most of them being very weak or weak, were not detrimental to the mixed model fits as the 12 selected items were the most available across participants and platforms (iOS, Android), and thus best representative of lifestyle and behaviors during this time.

Since backward elimination was applied to remove insignificant digital measure items from final models, sample sizes and the associated number of complete case digital measure sample observations used for the model fits may vary, so they are shown with the associated model below in the results section.

## Results

### Overview

Our main results consist of 17 mixed linear models. Note that the mixed model “Measures” (fixed-effects) such as “RAM” or “GPS Locations Visited” are described in the “Digital Measures” sections under Methods. Individual questions are examined here in addition to the total questionnaire score, as on their own, they were designed to capture various domains of mental health (e.g. depression, anxiety) [17] (discussed in Methods).

First, the demographics of the 94 participants in SHC 2.0 are presented. Then the results of the 17 mixed linear model fits are shown between the 12 selected phone digital measures and A) the SHC 2.0 daily questionnaire individual questions and total score, and B) the SHC 2.0 weekly questionnaire individual questions and total score. The results of the daily questionnaire model fits are presented to start as more data was collected daily. Note that weekly model fits and daily model fits are separate analyses designed for both infrequent and frequent responders, respectively. The preceding section Digital Measures–Overview describes how device measures get connected to both types questionnaire responses for the mixed model fits.

### Demographics

Table 2 presents the demographic information of the 94 participants in SHC 2.0. Participants were primarily male students living in off-campus housing.

**Table 2:** Participant demographics at baseline. As questions can be skipped, sometimes the cells for each item may not necessarily add to 100%.

		Participants (N = 94)	Percent %
**What is your gender?**			
	Female	22	23.4%
	Male	72	76.6%
**What degree are you completing?**			
	BA	34	36.2
	BEng	N/A	< = 5%
	BHealthSci	10	10.6
	BManagement	9	9.6
	BMedSci	16	17
	BMusic	N/A	< = 5%
	BSc	18	19.1
	BSc in Nursing	N/A	< = 5%
**What year are you currently in?**			
	First year	38	40.4
	Second year	20	21.3
	Third year	19	20.2
	Fourth year	12	12.8
	Fifth year+	5	5.3
**What is your current housing situation?**			
	Off-campus Apartment	10	10.6
	Off-campus House	65	69.1
	On-campus Residence	19	20.2

### Mixed linear models of SHC 2.0 daily questionnaire variant

**Fig 3:**
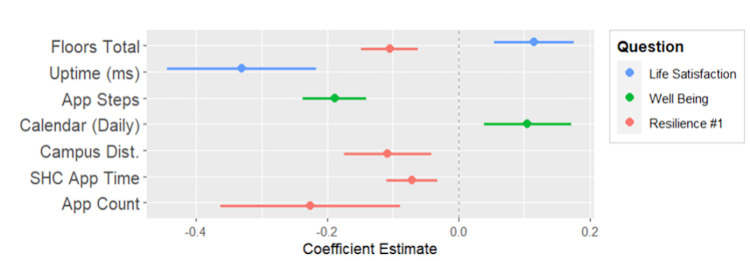
Coefficient plot for parameters of Daily Life Satisfaction, Daily Psychological Well-being and Daily Resilience Measure #1.

**Fig 4:**
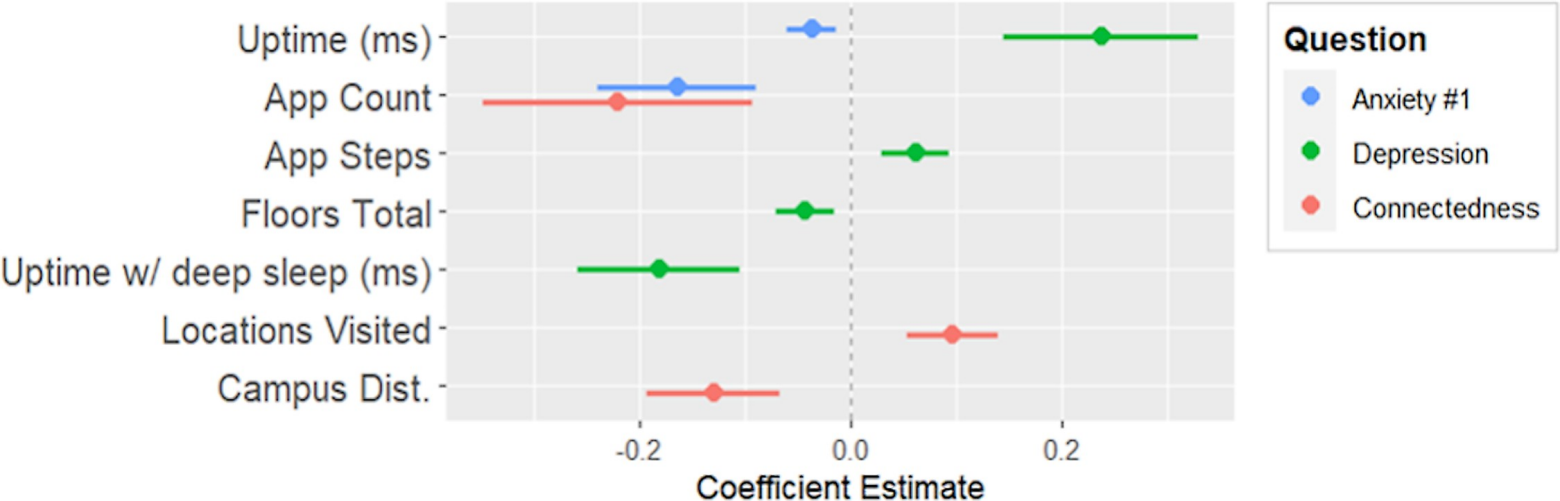
Coefficient plot for parameters of daily anxiety, daily depression, and daily community connectedness.

**Fig 5:**
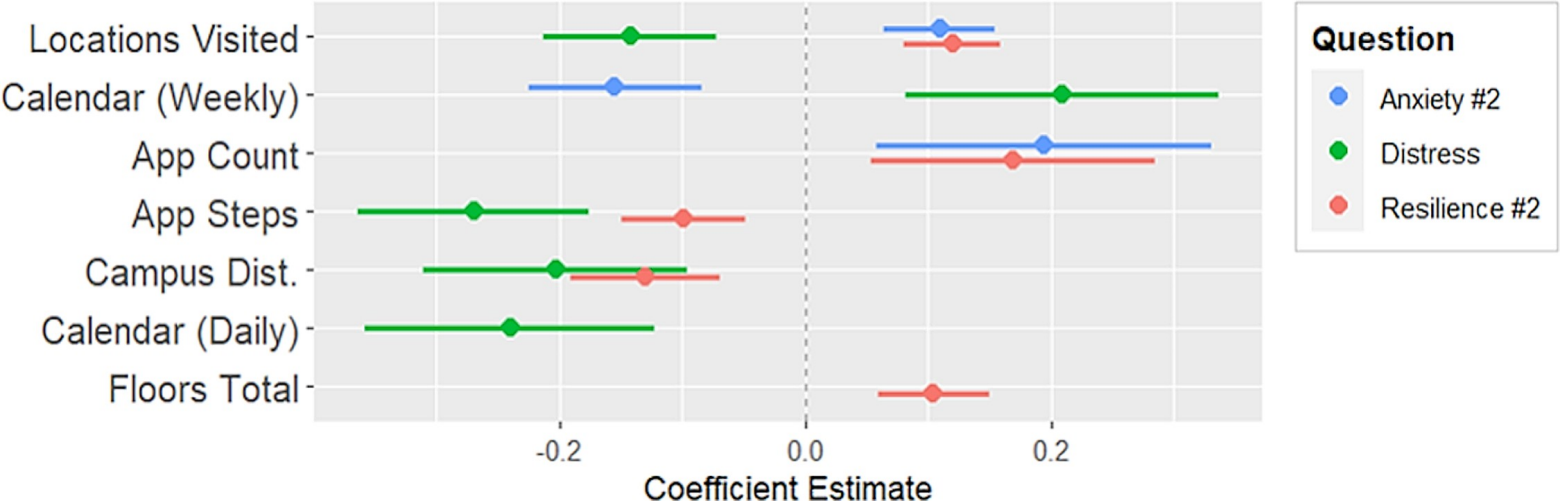
Coefficient plot for parameters of anxiety measure #2, distress, and resilience measure #2.

**Fig 6:**
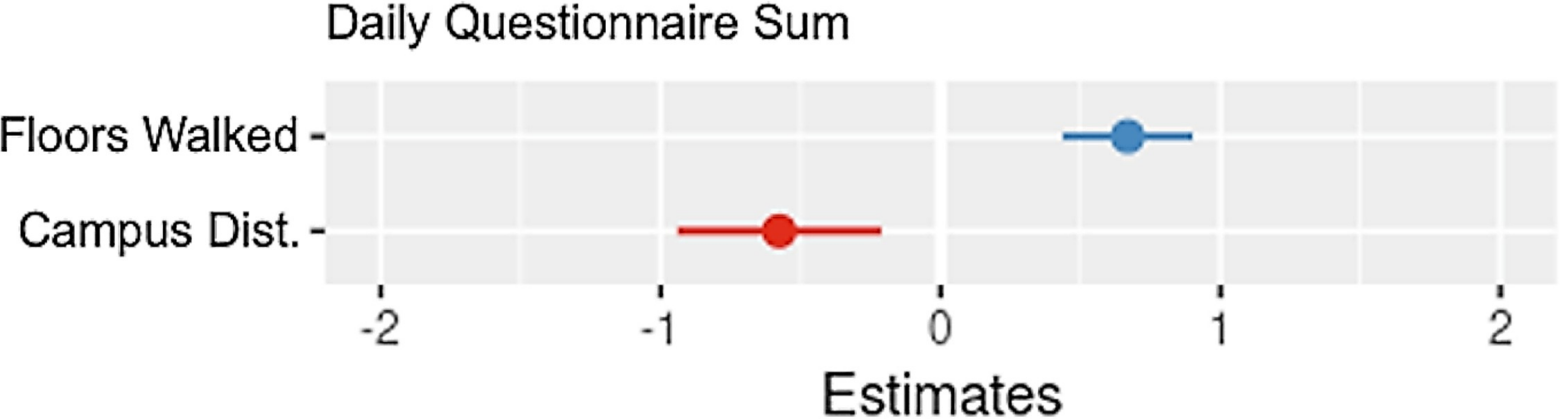
Coefficient plot for parameters of daily questionnaire sum.

In this section, the mixed linear model fits for the measures of the SHC 2.0 daily questionnaire are presented. Only the second round of model fits are shown here, which are fit using backward elimination of the insignificant effects of the first round of model fits. An explanation of how to interpret the tables and plots is found in S2 Appendix. Additionally, the first round is available in S2 Appendix in the same tabular format, and is also available as R output, found in S3 Appendix. The mixed model fits of questions 1–3 are found in Table 3 and Fig 3, questions 4–6 in Table 4 and Fig 4, and questions 7–9 in Table 5 and Fig 5. Finally, the total questionnaire sum mixed model fit is found in Table 6 and Fig 6.

**Table 3:** Mixed linear models for daily life satisfaction, daily psychological well-being, and daily resilience.

	Dependent variable		
	Life Satisfaction	Well-being	Resilience #1
Revised Model #	1	2	3
Observations	3356	3376	2514
Participants	58	58	46
Device digital measure	Parameter (SE)	Parameter (SE)	Parameter (SE)
Step count (weekly)		-0.189 (0.025)	
GPS locations visited			
Floors walked (up + down)	0.115 (0.031)		-0.105 (0.022)
Campus Dist. (less is closer to UWO)			-0.108 (0.034)
System uptime (ms)	-0.33 (0.058)		
Free space (percent)			
Time spent using SHC 2.0 app (ms)			-0.071 (0.020)
Calendar count (daily)		0.105 (0.034)	
Installed apps (total / List of 40)			-0.226 (0.070)

**Table 4:** Mixed linear models for daily anxiety, daily depression, and daily community connectedness.

	Dependent variable		
	Anxiety #1	Depression	Community Connectedness
Revised Model #	4	5	6
Observations	4573	2514	2514
Participants	73	46	46
Device digital measure	Parameter (SE)	Parameter (SE)	Parameter (SE)
Step count (weekly)		0.061 (0.016)	
GPS locations visited			0.096 (0.022)
Floors walked (up + down)		-0.044 (0.014)	
Campus Dist. (less is closer to UWO)			-0.130 (0.032)
System uptime (ms)	-0.037 (0.012)	0.237 (0.047)	
System uptime with deep sleep (ms)		-0.182 (0.039)	
Calendar count (weekly)			
Installed apps (total /List of 40)	-0.165 (0.038)		-0.221 (0.065)

**Table 5:** Mixed linear models for anxiety measure #2, distress, and resilience measure #2.

	Dependent variable		
	Anxiety #2	Distress	Resilience #2
Revised Model #	7	8	9
Observations	2514	2514	2494
Participants	46	46	46
Device digital measure	Parameter (SE)	Parameter (SE)	Parameter (SE)
Step count (weekly)		-0.27 (0.048)	-0.099 (0.026)
GPS locations visited	0.110 (0.023)	-0.142 (0.036)	0.12 (0.02)
Floors walked (up + down)			0.105 (0.023)
Campus Dist. (less is closer to UWO)		-0.203 (0.055)	-0.13 (0.031)
System uptime (ms)			
System uptime with deep sleep (ms)			
Free space (percent)			
Time spent using SHC 2.0 app (ms)			
Calendar count (daily)		-0.24 (0.060)	
Calendar count (weekly)	-0.155 (0.036)	0.21 (0.065)	
Installed apps (total /List of 40)	0.195 (0.0695)		0.169 (0.059)

**Table 6:** Revised second round (using only significant device digital measure items) of mixed linear model relating daily questionnaire sum to device digital measures. The first model is omitted for brevity. The initial model is included in S3 Appendix.

	Dependent variable
	Daily questionnaire variant sum
Revised Model #	10
Observations	2514
Participants	46
Device digital measure	Parameter (SE)
Floors walked (up + down)	0.669 (0.118)
Location flag	-0.576 (0.186)

#### Regressing daily questions 1–3 on device digital measures

System uptime had a negative association with life satisfaction. This suggests less device usage may be associated with life satisfaction. There also appeared to be a positive association with floors walked. Based on this model, physical activity did not appear to be associated with increased life satisfaction. Surprisingly, app steps had a negative association with daily psychological well-being. There were positive associations with locations visited (to an extent) and daily calendar counts, suggesting more life activity was related to psychological well-being. Floors walked, device usage via SHC 2.0 total app use time, and installed apps all had negative associations with the first daily measure of resilience. There also was a positive association with proximity to campus.

#### Regressing daily questions 4–6 on device digital measures

Uptime had a small negative association with the first measure of daily anxiety. There was a more significant negative association with the number of installed apps.

App steps had a minor positive association with daily depression. There was a positive association with system uptime and daily depression, and a negative association with system sleep time and depression. Locations visited, and proximity to campus had positive associations with daily community connectedness. There was a negative association with the number of installed apps with community connectedness.

#### Regressing daily questions 7–9 on device digital measures

Physical activity (locations visited) had a slight positive association with anxiety, while installed apps had a more positive association with anxiety. There was a negative association with weekly calendar events. Some measures of physical activity (app steps, locations visited) and daily calendar events had a negative association with distress. Proximity to campus and weekly calendar events had a positive association with distress. App steps had a slight negative association with the second daily measure of resilience. Locations visited, floors walked, proximity to campus, and installed app count all had positive associations with this measure of resilience.

#### Regressing daily total questionnaire sum on device digital measures

Floors walked and proximity to campus had positive associations with the daily questionnaire sum.

#### Mixed linear models of SHC 2.0 weekly questionnaire variant

**Fig 7:**
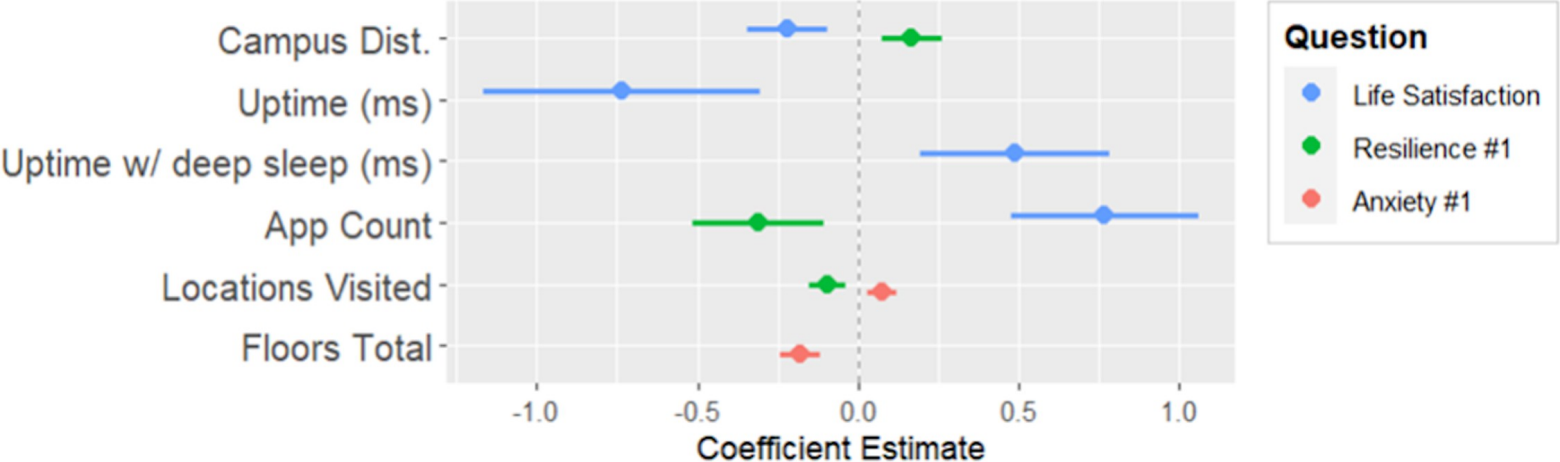
Coefficient plot for parameters of weekly life satisfaction, weekly resilience measure #1, weekly anxiety measure #1.

**Fig 8:**
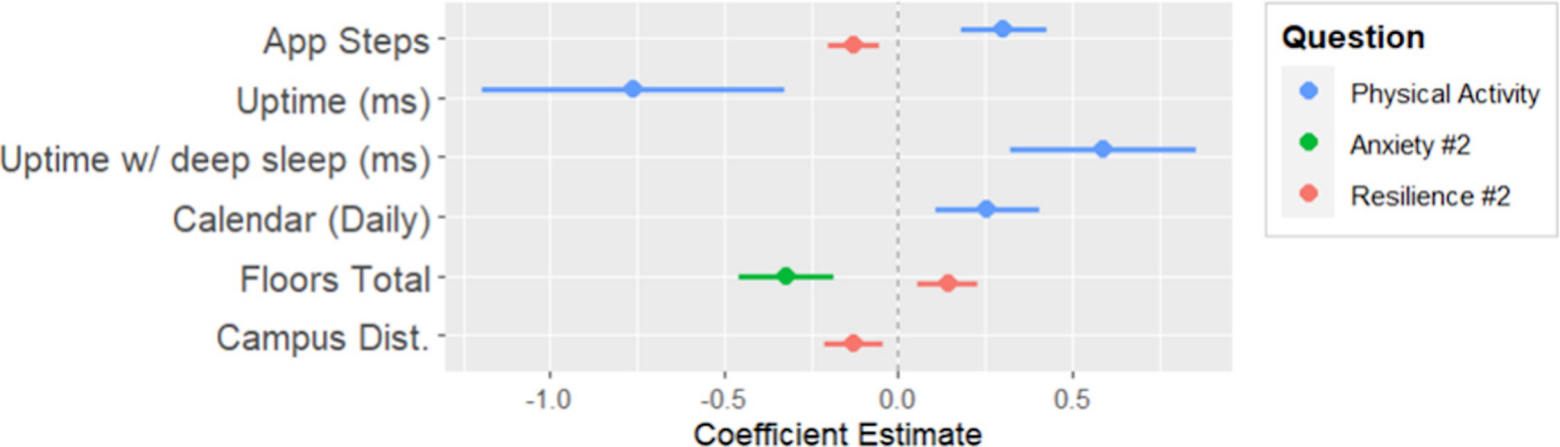
Coefficient plot for parameters of weekly physical activity, a second weekly anxiety measure, and a second weekly measure of resilience.

**Fig 9:**
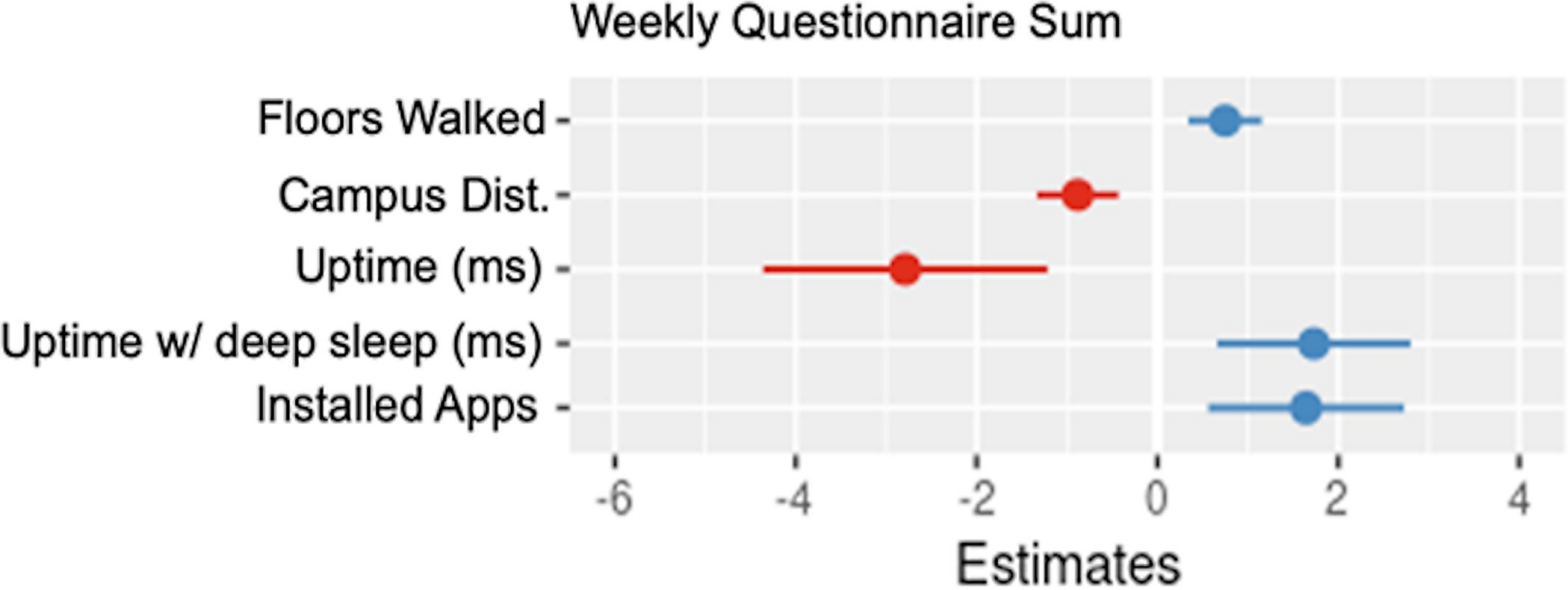
Coefficient plot for parameters of sum of weekly questionnaire.

In this section, model fits for the measures of the SHC 2.0 weekly questionnaire variant are presented. An explanation of how to interpret the tables and plots is found in S2 Appendix. Only the second round of model fits are shown here, which were fit using backward elimination of the insignificant effects of the first round, found in tabular format in S2 Appendix, and as R output in S3 Appendix. Note that for several weekly questions, mixed model fits did not yield any significant associations. This was the case for questions: 2 (psychological well-being), 5 (depression), and 6 (community connectedness). The mixed model fits for Life satisfaction, Resilience #1, and Anxiety #1, are in Table 7 and Fig 7. Mixed model fits for Physical Activity, Anxiety #2, and Resilience #2 are in Table 8 and Fig 8. Finally, the weekly total questionnaire sum mixed model fit is found in Table 9 and Fig 9.

**Table 7:** Mixed linear models for weekly life satisfaction, weekly resilience measure #1, weekly anxiety measure #1.

	Dependent variable		
	Life satisfaction	Resilience #1	Anxiety #1
Revised Model #	11	12	13
Observations	1003	992	1003
Participants	44	44	44
Device digital measure	Parameter (SE)	Parameter (SE)	Parameter (SE)
Step count (weekly)			
GPS locations visited		-0.093 (0.029)	0.075 (0.023)
Floors walked (up + down)			-0.180 (0.032)
Location flag	-0.221 (0.063)	0.167 (0.048)	
System uptime (ms)	-0.735 (0.220)		
System uptime with deep sleep (ms)	0.488 (0.151)		
Free space (percent)			
Calendar count (daily)			
Installed apps (total/List of 40)	0.769 (0.15)	-0.311 (0.103)	

**Table 8:** Mixed linear models for weekly physical activity, a second weekly anxiety measure, and a second weekly measure of resilience.

	Dependent variable		
	Physical Activity	Anxiety #2	Resilience #2
Revised Model #	14	15	16
Observations	1443	1443	1003
Participants	60	60	44
Device digital measure	Parameter (SE)	Parameter (SE)	Parameter (SE)
Step count (weekly)	0.303 (0.063)		-0.127 (0.038)
Floors walked (up + down)		-0.32163 (0.069)	0.143 (0.044)
Location flag			-0.129 (0.043)
System uptime (ms)	-0.762 (0.221)		
System uptime with deep sleep (ms)	0.589 (0.137)		
Calendar count (daily)	0.256 (0.077)		

**Table 9:** Mixed linear model for weekly questionnaire sum and device digital measures.

	Dependent variable
	Weekly questionnaire variant sum
Revised Model #	17
Observations	1003
Participants	44
Device digital measure	Parameter (SE)
Floors walked (up + down)	0.748 (0.207)
Campus Dist. (less is closer to UWO)	-0.881 (0.230)
System uptime (ms)	-2.789 (0.802)
System uptime with deep sleep (ms)	1.730 (0.545)
Installed apps (total/List of 40)	1.644 (0.553)

#### Regressing weekly questions 1, 3 and 4 on device digital measures

Proximity to campus and installed app count had positive associations with weekly life satisfaction. There was a positive association with system sleep time which may be indicative of participants getting more sleep. There was a corresponding negative association with system uptime. The first measure of weekly resilience had a positive association with distance from campus. Weekly resilience also had a negative association with installed app count and locations visited. There was a slight positive association with locations visited and weekly anxiety. This weekly anxiety measure had a negative association with floors walked.

#### Regressing weekly questions 7, 8, and 9 on device digital measures

As expected, app steps had a positive association with weekly physical activity. There was also a significant negative association in system up time, but a positive association with system sleep time, which may be indicative of physically active participants getting more sleep. Physically active participants had associations with more events on the day they completed the weekly survey. Like the first weekly anxiety measure, this measure of anxiety had a negative association with floors walked. App steps had a negative association with this second measure of weekly resilience. There was a positive association with floors walked, and proximity to campus.

#### Regressing weekly total questionnaire sum on device digital measures

Floors walked, proximity to campus, system sleep time and installed apps all had positive associations with the weekly questionnaire sum. System uptime had a negative association with the weekly questionnaire sum. These results may suggest participants were getting more sleep, despite the weekly score seemingly being associated with an increase in installed apps.

## Discussion

### Principal results

We previously summarized the results of the mixed models. The significant parameters of these models could be used in the development of new interventions.

While that is beyond the scope of this paper, some ideas for these future interventions might include text messages, information panels, or push notifications with content that might promote behavior aligning with the mixed model results shown here. More complicated interventions aiming for the same goal might resemble interactive virtual assistants that talk to users. Here we focus exclusively on any associations that might have existed between student psychosocial outcomes and day-to-day behavior and were not in a position to implement findings; future work could address this using our results. Additionally, to the best of our knowledge, there are no directly comparable works to SHC 2.0, but there are related ones. The most closely related Canadian work would be our COVID-specific study Student Pandemic Experience (SPE) [18,34] which had a larger sample size (315 participants), but the mental health questionnaires were tied to the pandemic, longer, and only done once per week. Globally, there is related work to SHC 2.0, but again, these studies do not appear to directly investigate associations between mental health measures and digital measures from hardware; the focus is elsewhere. As mentioned previously, our work was heavily influenced by Huckins et al [32]. They were able to compare a larger sample with pre-pandemic data, something that was not possible here. Furthermore, they investigated mental health measures with respect to news mentions which was not a research question here. Other recent comparable studies exist, but research questions tended to focus on mental health measures in relation to COVID rather than in relation to digital measures from hardware. Fried et al. [12] studied 80 undergraduates during the pandemic with EMA. Participants were sampled 4 times a day over 2 weeks, and the focus was on changes in lifestyle and mental health in relation to COVID measures. A study by Kleiman et al [13] had similarities but again focused on EMA data with respect to COVID. Schultz et al [14] and Arend et al [15] shared similar themes with being COVID-focused.

### Limitations

The models have some limitations which likely impacted the results. Missing complete case observations did vary on a weekly/daily basis which did limit the data used for our various mixed models. For instance, there were about 2500 complete case observations from 46 participants daily and 1000–1500 complete case observations from 40–60 participants weekly. We used complete case observations exclusively for this analysis since there were too many conditions to consider and values to impute for incomplete cases to integrate them into mixed model fits. We were unable to analyze call/text message data from participants due to increasing privacy restrictions on Android/iOS. The sample size is relatively small compared to the total population of a mid-size university in Canada, although there are a reasonable number of observations for the participants. Participants were nearly three quarters male students as well.

## Conclusions

Undergraduate studies (and pandemics) present unique conditions ‐ and opportunities ‐ for surveillance efforts with regards to mental health. This research found that there were some significant associations between phone digital measures and mental health-related self-reports from undergraduates in Ontario, Canada during pandemic conditions. While an initial analysis of our related SPE study data [34] appeared to produce more results as there were considerably more participants and digital measure samples (315 and approximately 25985, respectively) SPE was not designed as a long-term study and has concluded. There remains an opportunity to resume SHC 2.0 and compare data collected during the pandemic to post-pandemic data. Given that mental health will remain an issue of concern on university campuses, and to the general public in a broader sense, mental health stakeholders should consider the ongoing use of ethical, moment-by-moment surveillance techniques, such as the one presented here, to maximize data collection in order to make best informed decisions regarding mental health intervention design and potentially even individual mental health care. There are numerous opportunities to use technology like our EMAX platform to study day-to-day behavior, but even in a well-connected country like Canada, to the best of our knowledge, deployments of these ethically vetted EMA-type systems are rare. In this research, we were able to deploy an EMA-type system to observe behavioral associations with psychological measures of mental health, including anxiety, depression, and resilience. Overall, the SHC 2.0 study examined several measures of lifestyle, some of which appear to be associated with our selected measures of mental health in undergraduate students.

## Supporting information

S1 Appendix Smart Healthy Campus 2.0 App Questions. (DOCX)

S2 Appendix First round of mixed model fits for SHC 2.0. (DOCX)

S3 Appendix R output of SHC 2.0 mixed models, first round. (TXT)

S4 Appendix R output of SHC 2.0 mixed models, second round after backward elimination. (TXT)

## References

[1] Fried RR, Karmali S, Irwin JD, Gable FL, Salmoni A. Making the grade: Mentors’ perspectives of a course-based, smart, healthy campus pilot project for building mental health resiliency through mentorship and physical activity. Int J Evid Based Coach Mentor. 2018;16: 84–98. doi: 10.24384/000566

[2] Council of Ontario Universities. In It Together: Taking Action on Student Mental Health. 2017. Available: https://cou.ca/wp-content/uploads/2017/11/In-It-Together-PSE-Mental-Health-Action-Plan.pdf

[3] Doreleyers A, Knighton T. COVID-19 Pandemic: Academic Impacts on Postsecondary Students in Canada. StatCan COVID-19: Data to Insights for a Better Canada. Stat Canada. 2020.

[4] Wall K. COVID-19 Pandemic: financial impacts on postsecondary students in Canada. Stat Canada. 2020.

[5] Hicks LJ, Caron EE, Smilek D. SARS-CoV-2 and Learning: The Impact of a Global Pandemic on Undergraduate Learning Experiences. Scholarsh Teach Learn Psychol. 2021.

[6] Tasso AF, Hisli Sahin N, San Roman GJ. COVID-19 disruption on college students: Academic and socioemotional implications. Psychol Trauma Theory, Res Pract Policy. 2021. doi: 10.1037/tra0000996 33382329

[7] Prati G, Mancini AD. The psychological impact of COVID-19 pandemic lockdowns: A review and meta-analysis of longitudinal studies and natural experiments. Psychological Medicine. 2021. doi: 10.1017/S0033291721000015 33436130 PMC7844215

[8] Adlaf EM, Gliksman L, Demers A, Newton-Taylor B. The prevalence of elevated psychological distress among canadian undergraduates: Findings from the 1998 Canadian campus survey. J Am Coll Health Assoc. 2001. doi: 10.1080/07448480109596009 11590985

[9] Academic and Mental Health Needs of Students on a Canadian Campus. Can J Couns Psychother / Rev Can Couns psychothérapie. 2016.

[10] Giamos D, Lee AYS, Suleiman A, Stuart H, Chen S-P. Understanding Campus Culture and Student Coping Strategies for Mental Health Issues in Five Canadian Colleges and Universities. Can J High Educ. 2017. doi: 10.7202/1043242ar

[11] Wang R, Chen F, Chen Z, Li T, Harari G, Tignor S, et al. Studentlife: Assessing mental health, academic performance and behavioral trends of college students using smartphones. UbiComp 2014 ‐ Proceedings of the 2014 ACM International Joint Conference on Pervasive and Ubiquitous Computing. 2014. doi: 10.1145/2632048.2632054

[12] Fried EI, Papanikolaou F, Epskamp S. Mental Health and Social Contact During the COVID-19 Pandemic: An Ecological Momentary Assessment Study. Clin Psychol Sci. 2022. doi: 10.1177/21677026211017839

[13] Kleiman EM, Yeager AL, Grove JL, Kellerman JK, Kim JS. Real-time mental health impact of the COVID-19 pandemic on college students: ecological momentary assessment study. JMIR Ment Heal. 2020. doi: 10.2196/24815 33207308 PMC7744138

[14] Schulz PJ, Andersson EM, Bizzotto N, Norberg M. Using Ecological Momentary Assessment to Study the Development of COVID-19 Worries in Sweden: Longitudinal Study. J Med Internet Res. 2021. doi: 10.2196/26743 34847065 PMC8669580

[15] Arend AK, Blechert J, Pannicke B, Reichenberger J. Increased Screen Use on Days With Increased Perceived COVID-19-Related Confinements—A Day Level Ecological Momentary Assessment Study. Front Public Heal. 2021. doi: 10.3389/fpubh.2020.623205 33634062 PMC7902048

[16] Brogly C, Lizotte DJ, Bauer MA. Ecological Momentary Assessment eXtensions 3 (EMAX3) Proposal: An App for EMA-Type Research. 2021 Symposium on Computers and Communications (ISCC): 26th IEEE Symposium on Computers and Communications ‐ Workshop on ICT Solutions for eHealth (ICTS4eHealth) (ICTS4eHealth2021). Athens, Greece; 2021.

[17] Brogly C, Shoemaker JK, Lizotte DJ, Kueper JK, Bauer M. A Mobile App to Identify Lifestyle Indicators Related to Undergraduate Mental Health (Smart Healthy Campus): Observational App-Based Ecological Momentary Assessment. JMIR Form Res. 2021. doi: 10.2196/29160 34665145 PMC8564659

[18] Brogly C, Bauer MA, Lizotte DJ, Press ML, MacDougall A, Speechley M, et al. An app-based surveillance system for undergraduate students’ mental health during the covid-19 pandemic: Protocol for a prospective cohort study. JMIR Res Protoc. 2021. doi: 10.2196/30504 34516391 PMC8451963

[19] Mohr DC, Zhang M, Schueller SM. Personal Sensing: Understanding Mental Health Using Ubiquitous Sensors and Machine Learning. Annu Rev Clin Psychol. 2017. doi: 10.1146/annurev-clinpsy-032816-044949 28375728 PMC6902121

[20] Koo M, Skinner H. Challenges of internet recruitment: A case study with disappointing results. J Med Internet Res. 2005. doi: 10.2196/jmir.7.1.e6 15829478 PMC1550633

[21] Inglehart R., Haerpfer C., Moreno A., Welzel C., Kizilova K., Diez-Medrano J., Lagos M., Norris EP P.& BP et al. (eds.. World Values Survey: All Rounds ‐ Country-Pooled Datafile Version: http://www.worldvaluessurvey.org/WVSDocumentationWVL.jsp. Madrid: JD Systems Institute. In: World Values Survey: All Rounds ‐ Country-Pooled Datafile. 2014.

[22] Berwick DM, Murphy JM, Goldman PA, Ware JE, Barsky AJ, Weinstein MC. Performance of a five-item mental health screening test. Med Care. 1991. doi: 10.1097/00005650-199102000-00008 1994148

[23] Smith BW, Dalen J, Wiggins K, Tooley E, Christopher P, Bernard J. The brief resilience scale: Assessing the ability to bounce back. Int J Behav Med. 2008. doi: 10.1080/10705500802222972 18696313

[24] Spitzer RL, Kroenke K, Williams JBW, Löwe B. A brief measure for assessing generalized anxiety disorder: The GAD-7. Arch Intern Med. 2006. doi: 10.1001/archinte.166.10.1092 16717171

[25] Henry JD, Crawford JR. The short-form version of the Depression anxiety stress scales (DASS-21): Construct validity and normative data in a large non-clinical sample. Br J Clin Psychol. 2005. doi: 10.1348/014466505X29657 16004657

[26] Mashek D, Cannaday LW, Tangney JP. Inclusion of community in self scale: A single-item pictorial measure of community connectedness. J Community Psychol. 2007. doi: 10.1002/jcop.20146

[27] Milton K, Bull FC, Bauman A. Reliability and validity testing of a single-item physical activity measure. Br J Sports Med. 2011. doi: 10.1136/bjsm.2009.068395 20484314

[28] Ostroff JS, Woolverton KS, Berry C, Lesko LM. Use of the Mental Health Inventory with adolescents: A secondary analysis of the Rand Health Insurance Study. Psychol Assess. 1996. doi: 10.1037/1040-3590.8.1.105

[29] Heubeck BG, Neill JT. Confirmatory factor analysis and reliability of the mental health inventory for australian adolescents. Psychol Rep. 2000. doi: 10.2466/pr0.2000.87.2.431 11086588

[30] Sun S, Folarin AA, Ranjan Y, Rashid Z, Conde P, Stewart C, et al. Using smartphones and wearable devices to monitor behavioral changes during COVID-19. J Med Internet Res. 2020. doi: 10.2196/19992 32877352 PMC7527031

[31] Kuznetsova A, Brockhoff PB, Christensen RHB. lmerTest Package: Tests in Linear Mixed Effects Models. J Stat Softw. 2017. doi: 10.18637/jss.v082.i13

[32] Huckins JF, da Silva AW, Wang W, Hedlund E, Rogers C, Nepal SK, et al. Mental health and behavior of college students during the early phases of the COVID-19 pandemic: Longitudinal smartphone and ecological momentary assessment study. J Med Internet Res. 2020. doi: 10.2196/20185 32519963 PMC7301687

[33] Mack DL, DaSilva AW, Rogers C, Hedlund E, Murphy EI, Vojdanovski V, et al. Mental health and behavior of college students during the covid-19 pandemic: Longitudinal mobile smartphone and ecological momentary assessment study, part II. J Med Internet Res. 2021. doi: 10.2196/28892 33900935 PMC8183598

[34] Brogly C. Observations of positive mental health indicators in undergraduates using specialized mobile apps during the COVID-19 pandemic. Western University. 2022. Available: https://ir.lib.uwo.ca/etd/8532

