# Supplementary material for: An app-based ecological momentary assessment of undergraduate student mental Health during the COVID-19 pandemic in Canada (Smart Healthy Campus Version 2.0): Longitudinal study
Source: PLOS Digit Health. 2024 May 20;3(5):e0000239. doi: 10.1371/journal.pdig.0000239 (PMC11104666; doi:10.1371/journal.pdig.0000239)
Supplement: S2 Appendix — (DOCX) [file pdig.0000239.s002.docx]

#### S2 Appendix: First round of mixed model fits for SHC 2.0

#### Interpretation of coefficient plots

This section explains how to interpret the coefficient plots of the mixed models below. Every plot shows the amount of change associated with the dependent variable (questionnaire measure) as there is a +1 unit change in the digital measure item (independent variable, y-axis). Note that digital measure items were z-score standardized due to different very scales, so a +1 unit change is a +1 standard deviation from the mean. Dependent variables were not standardized for future prediction purposes. Essentially, an association can be positive or negative, and the value on the x-axis “Estimates” for any predictor on the y-axis would be added to the dependent variable as there is a +1 unit change for that y-axis predictor.

Table 1. First round of mixed linear models for daily life satisfaction, daily psychological well-being, and daily resilience.

|  | Dependent variable | | | | | |
| --- | --- | --- | --- | --- | --- | --- |
|  | Daily Q1 |  | Daily Q2 |  | Daily Q3 |  |
| Measure | Life Satisfaction |  | Well-being |  | Resilience #1 |  |
| Model # | 1 |  | 2 |  | 3 |  |
| Observations | 2494 |  | 2494 |  | 2494 |  |
| Participants | 46 |  | 46 |  | 46 |  |
|  |  |  |  |  |  |  |
| Device digital measure | Parameter (SE) | P-value | Parameter (SE) | P-value | Parameter (SE) | P-value |
| Step count  (weekly) | -0.079 (0.046) | 0.088 | -0.185(0.034) | 4.2E-08 | -0.001(0.031) | 0.958 |
| GPS locations visited | -0.059 (0.036) | 0.105 | 0.068 (0.026) | 0.009 | -0.004(0.025) | 0.861 |
| Floors walked (up + down) | 0.24 (0.041) | 7.67E-09 | -0.065 (0.03) | 0.029 | -0.107 (0.028) | 0.000 |
| Campus Dist. (less is closer to UWO) | -0.0915 (0.055) | 0.098 | 0.050 (0.04) | 0.206 | -0.099 (0.037) | 0.008 |
| System uptime (ms) | -0.989 (0.147) | 2.14E-11 | 0.154 (0.105) | 0.144 | -0.026 (0.099) | 0.786 |
| System uptime with deep sleep (ms) | 0.546 (0.116) | 0.000 | -0.051 (0.083) | 0.532 | 0.071 (0.078) | 0.359 |
| Available RAM (bytes) | -0.023 (0.089) | 0.793 | -0.036 (0.064) | 0.573 | 0.039 (0.060) | 0.514 |
| Free space  (percent) | -0.323 (0.082) | 0.000 | 0.036 (0.057) | 0.536 | 0.029 (0.054) | 0.591 |
| Time spent using SHC 2.0 app (ms) | 0.079 (0.033) | 0.016 | -0.023 (0.024) | 0.342 | -0.071 (0.022) | 0.001 |
| Calendar count (daily) | -0.085 (0.057) | 0.139 | 0.129 (0.041) | 0.001 | 0.022 (0.039) | 0.565 |
| Calendar count (weekly) | 0.019 (0.063) | 0.758 | -0.063 (0.045) | 0.160 | 0.023 (0.042) | 0.597 |
| Installed apps (total /  List of 40) | 0.009 (0.107) | 0.932 | -0.105 (0.076) | 0.163 | -0.232 (0.071) | 0.001 |

Table 2. First round of mixed linear models for daily anxiety, daily depression, and daily community connectedness.

|  | Dependent variable | | | | | |
| --- | --- | --- | --- | --- | --- | --- |
|  | Daily Q4 |  | Daily Q5 |  | Daily Q6 |  |
| Measure | Anxiety #1 |  | Depression |  | Comm. Connected. |  |
| Model # | 4 |  | 5 |  | 6 |  |
| Observations | 2494 |  | 2494 |  | 2494 |  |
| Participants | 46 |  | 46 |  | 46 |  |
|  |  |  |  |  |  |  |
| Device digital measure | Parameter (SE) | P-value | Parameter (SE) | P-value | Parameter (SE) | P-value |
| Step count  (weekly) | -0.04(0.021) | 0.069 | 0.061 (0.016) | 0.000 | 0.054 (0.028) | 0.057 |
| GPS locations visited | 0.039 (0.017) | 0.022 | 0.026 (0.013) | 0.049 | 0.097 (0.022) | 0.000 |
| Floors walked (up + down) | -0.051 (0.019) | 0.007 | -0.047 (0.014) | 0.001 | 0.0041 (0.025) | 0.871 |
| Campus Dist. (less is closer to UWO) | -0.022 (0.025) | 0.382 | 0.050 (0.019) | 0.011 | -0.127 (0.034) | 0.000 |
| System uptime (ms) | 0.237 (0.068) | 0.000 | 0.254 (0.052) | 0.000 | 0.218 (0.090) | 0.017 |
| System uptime with deep sleep (ms) | -0.067 (0.053) | 0.214 | -0.193 (0.042) | 0.000 | -0.208 (0.072) | 0.004 |
| Available RAM (bytes) | 0.023 (0.041) | 0.564 | -0.008 (0.031) | 0.789 | -0.0545 (0.054) | 0.321 |
| Free space  (percent) | -0.062 (0.038) | 0.105 | 0.021 (0.031) | 0.498 | 0.072 (0.051) | 0.157 |
| Time spent using SHC 2.0 app (ms) | 0.015 (0.015) | 0.328 | -0.013 (0.011) | 0.276 | -0.035 (0.020) | 0.085 |
| Calendar count (daily) | 0.011 (0.026) | 0.686 | 0.006 (0.021) | 0.771 | -0.061 (0.036) | 0.084 |
| Calendar count (weekly) | -0.015 (0.029) | 0.622 | -0.016 (0.022) | 0.483 | 0.104 (0.039) | 0.008 |
| Installed apps (total /  List of 40) | -0.148 (0.049) | 0.003 | -0.045 (0.039) | 0.255 | -0.225 (0.066) | 0.000 |

Table 3. First round of mixed linear models for daily anxiety, distress, and resilience.

|  | Dependent variable | | | | | |
| --- | --- | --- | --- | --- | --- | --- |
|  | Daily Q7 |  | Daily Q8 |  | Daily Q9 |  |
| Measure | Anxiety #2 |  | Distress |  | Resilience # 2 |  |
| Model # | 7 |  | 8 |  | 9 |  |
| Observations | 2494 |  | 2494 |  | 2494 |  |
| Participants | 46 |  | 46 |  | 46 |  |
|  |  |  |  |  |  |  |
| Device digital measure | Parameter (SE) | P-value | Parameter (SE) | P-value | Parameter (SE) | P-value |
| Step count  (weekly) | 0.099 (0.031) | 0.001 | -0.266 (0.049) | 7.11E-08 | -0.1 (0.026) | 0.000 |
| GPS locations visited | 0.087 (0.024) | 0.000 | -0.134 (0.038) | 0.000 | 0.122 (0.021) | 3.7E-09 |
| Floors walked (up + down) | 0.057 (0.028) | 0.039 | 0.089 (0.044) | 0.041 | 0.107 (0.023) | 0.000 |
| Campus Dist. (less is closer to UWO) | -0.085 (0.036) | 0.022 | -0.201 (0.057) | 0.000 | -0.131 (0.031) | 0.000 |
| System uptime (ms) | 0.027 (0.099) | 0.784 | 0.111 (0.154) | 0.470 | 0.19 (0.083) | 0.023 |
| System uptime with deep sleep (ms) | 0.113 (0.077) | 0.146 | -0.133 (0.12) | 0.267 | -0.167 (0.065) | 0.01 |
| Available RAM (bytes) | 0.012 (0.06) | 0.84 | -0.022 (0.094) | 0.815 | -0.01 (0.05) | 0.838 |
| Free space  (percent) | 0.059 (0.053) | 0.271 | 0.031 (0.08) | 0.693 | -0.091 (0.045) | 0.045 |
| Time spent using SHC 2.0 app (ms) | -0.056 (0.022) | 0.012 | 0.064 (0.035) | 0.067 | -0.05 (0.018) | 0.006 |
| Calendar count (daily) | 0.028 (0.038) | 0.462 | -0.243 (0.06) | 5.85E-05 | -0.05 (0.033) | 0.124 |
| Calendar count (weekly) | -0.17 (0.042) | 0.000 | 0.207 (0.07) | 0.001 | 0.023 (0.036) | 0.52 |
| Installed apps (total /  List of 40) | 0.192 (0.07) | 0.006 | 0.07 (0.105) | 0.502 | 0.17 (0.06) | 0.004 |

Table 4. First round of mixed linear models for weekly life satisfaction, weekly resilience, and weekly anxiety.

|  | Dependent variable | | | | | |
| --- | --- | --- | --- | --- | --- | --- |
|  | Weekly Q1 |  | Weekly Q3 |  | Weekly Q4 |  |
|  | Life Satisfaction |  | Resilience #1 |  | Anxiety #1 |  |
| Model # | 11 |  | 12 |  | 13 |  |
| Observations | 992 |  | 992 |  | 1003 |  |
| Participants | 44 |  | 44 |  | 44 |  |
|  |  |  |  |  |  |  |
| Device digital measure | Parameter (SE) | P-value | Parameter (SE) | P-value | Parameter (SE) | P-value |
| Step count  (weekly) | 0.107 (0.058) | 0.066 | 0.0683 (0.041) | 0.096 | 0.022 (0.032) | 0.498 |
| GPS locations visited | 0.014 (0.043) | 0.747 | -0.098 (0.03) | 0.001 | 0.077 (0.023) | 0.001 |
| Floors walked (up + down) | 0.032 (0.065) | 0.620 | -0.113 (0.046) | 0.013 | -0.182 (0.035) | 3.31E-07 |
| Campus Dist. (less is closer to UWO) | -0.258 (0.07) | 0.000 | 0.184 (0.049) | 0.000 | 0.075 (0.04) | 0.048 |
| System uptime (ms) | -0.696 (0.245) | 0.004 | 0.193 (0.172) | 0.259 | 0.022 (0.133) | 0.865 |
| System uptime with deep sleep (ms) | 0.461 (0.165) | 0.005 | -0.044 (0.116) | 0.707 | -0.073 (0.089) | 0.412 |
| Available RAM (bytes) | 0.072 (0.102) | 0.478 | 0.064 (0.072) | 0.37 | -0.062 (0.055) | 0.260 |
| Free space  (percent) | -0.173 (0.168) | 0.304 | 0.303 (0.1171) | 0.01 | 0.061 (0.085) | 0.471 |
| Time spent using SHC 2.0 app (ms) | -0.036 (0.067) | 0.589 | -0.014 (0.047) | 0.763 | 0.059 (0.036) | 0.108 |
| Calendar count (daily) | 0.164 (0.064) | 0.011 | 0.012 (0.045) | 0.786 | -0.039 (0.035) | 0.269 |
| Calendar count (weekly) | -0.152 (0.109) | 0.163 | 0.008 (0.076) | 0.916 | -0.093 (0.059) | 0.115 |
| Installed apps (total /  List of 40) | 0.766 (0.155) | 0.000 | -0.369 (0.108) | 0.000 | -0.182 (0.08) | 0.025 |

Table 5. First round of mixed linear models for weekly physical activity, weekly anxiety, and weekly resilience.

|  | Dependent variable | | | | | |
| --- | --- | --- | --- | --- | --- | --- |
|  | Weekly Q7 |  | Weekly Q8 |  | Weekly Q9 |  |
|  | Physical Activity |  | Anxiety #2 |  | Resilience #2 |  |
| Model # | 14 |  | 15 |  | 16 |  |
| Observations | 1443 |  | 1443 |  | 1003 |  |
| Participants | 60 |  | 60 |  | 44 |  |
|  |  |  |  |  |  |  |
| Device digital measure | Parameter (SE) | P-value | Parameter (SE) | P-value | Parameter (SE) | P-value |
| Step count  (weekly) | 0.384 (0.082) | 3.15E-06 | -0.049 (0.087) | 5.77E-01 | -0.117 (0.040) | 4.08E-03 |
| GPS locations visited | 0.032 (0.059) | 0.585 | -0.04 (0.063) | 0.525 | -0.027 (0.029) | 0.357 |
| Floors walked (up + down) | 0.091 (0.091) | 0.317 | -0.356 (0.097) | 0.000 | 0.146 (0.045) | 0.001 |
| Campus Dist. (less is closer to UWO) | 0.076 (0.095) | 0.423 | 0.09 (0.095) | 0.347 | -0.105 (0.047) | 0.026 |
| System uptime (ms) | -0.949 (0.334) | 0.004 | 0.196 (0.334) | 0.557 | 0.41 (0.167) | 0.014 |
| System uptime with deep sleep (ms) | 0.588 (0.224) | 0.0089 | -0.027 (0.221) | 0.901 | -0.217 (0.112) | 0.054 |
| Available RAM (bytes) | -0.052 (0.144) | 0.718 | 0.1 (0.158) | 0.526 | 0.007 (0.071) | 0.915 |
| Free space  (percent) | 0.497 (0.191) | 0.011 | -0.138 (0.147) | 0.352 | 0.061 (0.1) | 0.54 |
| Time spent using SHC 2.0 app (ms) | -0.038 (0.094) | 0.681 | 0.026 (0.1) | 0.795 | -0.099 (0.046) | 0.034 |
| Calendar count (daily) | 0.347 (0.09) | 0.000 | -0.257 (0.096) | 0.007 | 0.053 (0.045) | 0.235 |
| Calendar count (weekly) | -0.334 (0.148) | 0.024 | 0.188 (0.146) | 0.198 | -0.164 (0.074) | 0.026 |
| Installed apps (total /  List of 40) | -0.134 (0.189) | 0.478 | -0.124 (0.161) | 0.441 | -0.18 (0.097) | 0.065 |
